# Uveitis as a Predictor of Predisposition to Autoimmunity

**DOI:** 10.7759/cureus.7451

**Published:** 2020-03-28

**Authors:** Jillian A Leibowitz, Arden T Woods, Marc M Kesselman, Bindu S Mayi

**Affiliations:** 1 Osteopathic Medicine, Dr. Kiran C. Patel College of Osteopathic Medicine, Nova Southeastern University, Davie, USA; 2 Rheumatology, Dr. Kiran C. Patel College of Osteopathic Medicine, Nova Southeastern University, Davie, USA; 3 Basic Sciences, Dr. Kiran C. Patel College of Osteopathic Medicine, Nova Southeastern University, Davie, USA

**Keywords:** uveitis, vaccine, adverse reaction, autoimmunity, autoimmune flare-up

## Abstract

The right environmental trigger can lead to immune system activation, which, in turn, can create an autoimmune reaction. Although each autoimmune disease is characterized by specific symptoms, many nonspecific symptoms can make these conditions difficult to diagnose. In this literature review, we seek an association between immunization-induced uveitis and an autoimmune diagnosis and/or autoimmune flare-up in patients. Our goal is to consider adverse reactions to vaccines as a possible warning sign of current or future autoimmune disease. If an immunization-induced adverse reaction is known to be a predictor of an autoimmune disease, the clinician could raise suspicion for autoimmune disease when a patient presents with vaccine-associated uveitis. While no direct correlations can be made yet, our review supports closer scrutiny of the association of immunizations and autoimmune disorders. The occurrence of uveitis across several autoimmune diseases could mean a possible link between vaccine-induced uveitis and undiagnosed autoimmune disease. Researchers can, therefore, perform retrospective studies on vaccinated patients and investigate the occurrence of uveitis, along with the timeframe of resolution and presenting symptoms at the time of the diagnosis of autoimmune disease.

## Introduction and background

Discovering possible links between vaccines and autoimmune diseases has been an area of inquiry for researchers for many years, but previous studies have largely focused on cause-and-effect relationships [1]. Autoimmune diseases are immune-mediated attacks on body tissues resulting from the loss of self-tolerance within the immune system. With a high prevalence in women [2], the underlying cause is thought to be a mix of environmental triggers and genetic susceptibility [3]. Many autoimmune diseases have an association with specific human leukocyte antigen (HLA) types. For example, spondyloarthropathies have a strong association with HLA-B27 [4], while HLA-DR4 shows a higher incidence with rheumatoid arthritis [5]. The right environmental trigger can lead to immune system activation, epitope spreading, bystander activation, molecular mimicry of foreign antigens to self-peptides, and/or the autoactivation of B and T cells, all of which can incite an autoimmune reaction [6]. Gut dysbiosis, microbial infections, toxic chemical exposures, and dietary components are just some of the many potential environmental triggers to aggravate an autoimmune disease [7]. Some of the more prevalent autoimmune diseases include rheumatoid arthritis, systemic lupus erythematosus, scleroderma, and Sjogren’s syndrome [8]. Although each disease is characterized by specific symptoms, many overlap with generally nonspecific symptoms, such as fatigue, malaise, swelling/redness, muscle aches, pains, low-grade fever, and rash, making these conditions difficult to diagnose [9]. 

Vaccines are administered antigenic materials that activate the immune system to develop adaptive immunity to specific pathogens. The purpose of vaccinations is to ultimately reduce the prevalence of infections amongst a larger population [10]. Vaccines are classified as live attenuated, killed/inactivated, recombinant/subunit, or toxoid type [11]. Research has shown that patients with autoimmune diseases can be administered inactivated vaccinations regardless of their treatment protocol. However, in regards to live vaccinations, patients taking biological medications, disease-modifying anti-rheumatic drugs, and glucocorticoids need to take caution due to immunosuppression and are recommended against live attenuated vaccinations at this time [12]. 

Killed vaccines contain inactivated pathogens that maintain the epitope structure on the surface, enabling it to induce a humoral response. Examples of inactivated vaccines include those for rabies, influenza (shot only), polio (shot only), and hepatitis A [11]. Live attenuated vaccines induce both cellular and humoral responses. Examples of live vaccines include those for varicella, smallpox, yellow fever, influenza (intranasal), measles, mumps, and rubella (MMR), and rotavirus [11, 13]. Many vaccines now contain adjuvants that are used to boost the immune system and create a more effective response in the body. The different types of adjuvants include aluminum combinations with salts, hydroxide, phosphate, and potassium sulfate [14].

Although adjuvants are mixed into the vaccine, they do not form stable linkages with the antigen. Adjuvants work by increasing the production of local inflammatory cytokines which will activate immune cells and their pattern recognition receptors (PRR). In addition, adjuvants improve antigen delivery, processing, and presentation by antigen-presenting cells [15]. One example of a widely used adjuvant is aluminum salts, which cause the vaccine antigen to precipitate, allowing slower release, and greater exposure time, as well as strengthen antigen uptake by antigen-presenting cells [16]. 

Uveitis is an inflammatory reaction of the uvea - the middle tissue layer of the eyewall composed of the iris, ciliary body, and choroid. Symptoms include redness, photosensitivity, blurriness, and eye pain. There are several types of uveitis depending on the site of inflammation. Anterior uveitis, the most common form, presents as iritis [17]. Posterior uveitis manifests as choroiditis and retinitis, and diffuse or panuveitis consists of all eyewall layers being affected [17].

Patients can present with infectious, noninfectious, and drug-induced uveitis. Uveitis can also be associated as a common systemic manifestation of autoimmune diseases, such as sarcoidosis, systemic lupus erythematosus, rheumatoid arthritis, Reiter’s syndrome, certain HLA-B27-associated diseases, as well as extra-articular manifestations of spondyloarthropathies, ankylosing spondylitis, psoriatic arthritis, and inflammatory bowel disease [18-19]. Spondylarthritis is the leading cause of uveitis in Western countries [20]. One study showed that about two-thirds of patients are also diagnosed with a specific joint disease while being evaluated for anterior uveitis [20]. Oftentimes, uveitis is overlooked and is thereby underdiagnosed [21]. Aside from drug-induced uveitis, an infrequently diagnosed uveitis is an adverse effect of vaccinations [17]. In this literature review, we look for immunization-induced uveitis preceding an autoimmune diagnosis and/or autoimmune flare-up in patients.

Vaccinations, including hepatitis B, human papillomavirus, the Bacille Calmette-Guerin (BCG) vaccine for tuberculosis, Shingrix®, and influenza, among others, have revealed individuals at risk for developing uveitis associated with an autoimmune disease that the patient has been unaware of prior to immunization [17, 22]. The purpose of this article is to determine whether we can use the occurrence of ocular side effects of vaccinations as a predictor of autoimmune disease since uveitis has been documented as an adverse reaction following several vaccinations [17].

## Review

**Figure 1:**
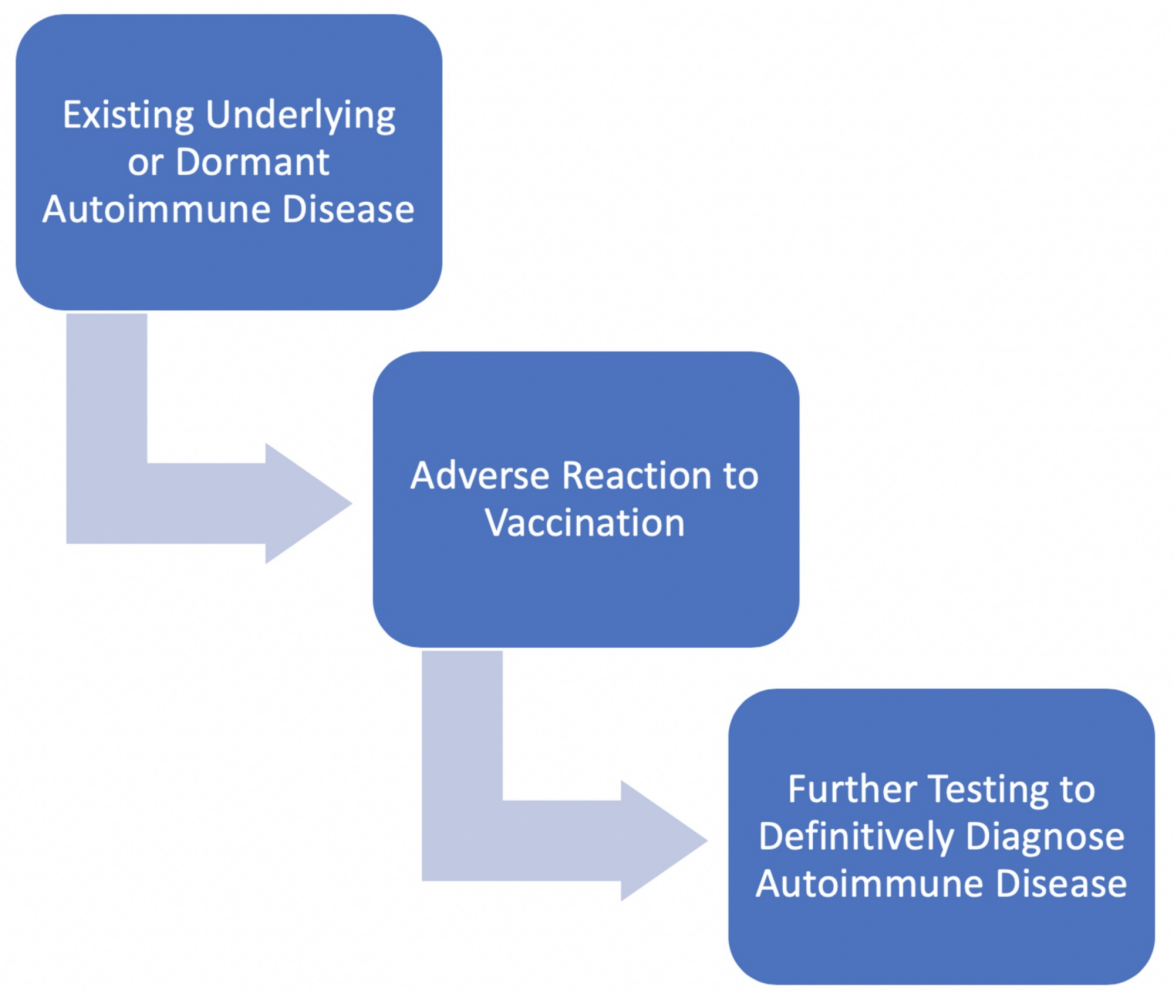
The proposed diagnostic process for discovery of an autoimmune disease based on the presentation of an adverse vaccine reaction A patient with an existing undiagnosed or dormant autoimmune disease develops a vaccine-associated adverse reaction, such as uveitis or a localized lupus reaction, which can alert the healthcare provider to perform further testing leading to the discovery of the previously unrecognized autoimmune disease.

Many studies have concentrated on large populations and their future risk for autoimmune diseases after vaccination [23]. However, the purpose of this paper is to examine a much smaller subset of that population. It is not to establish a cause-and-effect relationship but to consider adverse reactions to vaccines as a possible warning sign of current or future autoimmune disease. If this link is established, a clinician could raise their index of suspicion for autoimmune disease when a patient presents with vaccine-associated uveitis (as shown in Figure 1). Here, we will analyze research to explore how patients presenting with adverse reactions from a recent vaccination may be a part of the same population that already has an undiagnosed autoimmune disease or will develop autoimmune disease in the future for reasons unrelated to the vaccination. To do this, we propose molecular mimicry, an immune reaction to vaccines, human leukocyte antigens (HLA), and delayed hypersensitivity reaction with immune complex deposition to be associated with adverse reactions to vaccines and autoimmune disease.

Uveitis has been documented as a reaction following vaccinations with several different vaccines, including those for hepatitis B, human papillomavirus, influenza, BCG, MMR, varicella, as well as the simultaneous administration of multiple vaccines [17]. Authors of one study hypothesized the origin of vaccine-induced uveitis to result from one of three mechanisms: molecular mimicry of the vaccine peptide fragments to uveal peptides, a delayed hypersensitivity reaction to the vaccine with the involvement of immune complex deposition, or a direct immune reaction to an antigen (the vaccine adjuvants) [17]. All three of these processes are independently recognized as pathologic mechanisms in the development of autoimmune diseases [24]. A thought here is that a patient who experiences molecular mimicry, immune complex deposition, and generalized immune reactions from one source (an autoimmune disease) may be more likely to experience it from another source (vaccines) regardless of whether the autoimmune disease has manifested clinically at the time of reaction to the vaccine, much in the way that patients with one autoimmune disease often present with other autoimmune diseases [25]. 

To this end, studies have been performed that describe the appearance of autoimmune diseases after vaccination, some with antecedent adverse reactions to the vaccination [22, 26-28]. One such case study described the appearance of uveitis sarcoidosis four days after the administration of the Shingrix vaccine in a 53-year-old patient with no prior history of sarcoidosis [22]. Subsequent lab tests found the patient to have elevated levels of 1,25-OH-vitamin D levels, angiotensinogen-converting enzyme (ACE), rheumatoid factor, and calcium ions indicating the presence of an autoimmune disease. Interestingly, after treatment with corticosteroids, the ACE level returned to normal levels and her symptoms resolved. After cessation of treatment, symptoms reoccurred in her right eye which then responded to ophthalmic corticosteroid medication. This medication was still being administered at the time of publication of the article for maintenance treatment. Authors of the article hypothesize that the Shringrix vaccination aggravated a “dormant” autoimmune disease through the processes of molecular mimicry and induction of the immune system. There is ongoing research regarding the Shingrix vaccine specifically and whether it should be considered contraindicated in patients with autoimmune disease. While there is no definitive answer to the origin of the uveitis sarcoidosis, this case introduces the possibility that adverse reactions to vaccines could provide positive predictive power to the existence of an underlying autoimmune disease. 

In a similar case, an 18-month-old boy developed ulcerated lupus vulgaris at the puncture site of his BCG vaccination [26]. Like in the last case, the lesion resolved with treatment which, in this case, was isoniazid and rifampicin. Unfortunately, it is not known whether the child later developed lupus or another autoimmune disorder. However, another study described the development of lupus vulgaris in three patients, years after they originally presented with the same lesion that the 18-month-old boy developed following the same BCG vaccination [27]. Together, these studies corroborate our hypothesis that adverse reactions to vaccines may be a warning sign of underlying immune disease.

Technological advances have enhanced the delivery of medicine. Also, as we move forward with computerized medicine and electronic medical records, the patient history can be shared amongst specialties, improving chances of garnering minor details that could potentially save a patient’s life or discover the appropriate diagnosis sooner. For instance, a patient may present to ophthalmology with ocular discomfort. With proper treatment, the patient may resolve the ophthalmic symptom, but a potential systemic manifestation may go unnoticed without referral to rheumatology. Ocular manifestations are typical findings among systemic autoimmune diseases [29]. An analytical review of 300 rheumatology consultations from a Veterans Administration healthcare system revealed that about 4% of cases were referred to rheumatology by ophthalmologists due to the high prevalence of anterior uveitis and keratoconjunctivitis sicca as potential rheumatic diseases needing a more extensive workup [30]. 

We want to promote awareness among patients and physicians about vaccine-associated uveitis resulting from an underlying, unnoticed, and undiagnosed autoimmune disorder. Dating back to 1984, there have been reported cases of vaccine-induced uveitis. This review was limited by the lack of patient history and covered case reports over 30 years. Researchers found 289 vaccine-related uveitis cases due to vaccinations with BCG, Brucella, diphtheria-pertussis-tetanus, hepatitis A, hepatitis B, human papillomavirus (HPV), influenza, MMR, pneumococcal, pox viral particles, smallpox, tetanus, polio, and varicella. However, a major setback of this review was the lack of patient history which could have revealed underlying diseases or associations with rheumatic diseases. The review did not disclose whether the polio vaccine was live or inactivated, which may have influenced the results. Additionally, this review did not report the clinical features of each patient’s uveitis presentation. Despite that, the review reveals the hepatitis B vaccine as the most frequent cause of vaccine-associated uveitis [17].

Another link between adverse vaccine reactions and their ability to predict autoimmune disease is the existence of “high-risk” HLA proteins in some of the vaccine-induced presentations analyzed here. It has been known for some time that heritable HLA proteins, such as HLA-B27, HLA-DR2, HLA-DR3, and HLA-DR4, have been implicated in the development of autoimmune diseases, such as ankylosing spondylitis and systemic lupus erythematosus [31-33]. However, more recently, studies have linked some of these same HLA proteins with the incidence of adverse reactions to vaccines [28]. The fact that these HLA proteins are associated with both autoimmune diseases and adverse vaccine reactions could prove useful clinically. It could even help providers diagnose autoimmune diseases earlier for certain patients, such as the one presented in the following study [34]. 

One case study described in the literature is of chronic bilateral anterior uveitis after administration of the BCG vaccine in a 13-year-old girl who was positive for HLA-DR4 (a protein implicated in the development of rheumatoid arthritis and type one diabetes) [34]. Much like the cases discussed earlier, the mechanism proposed for this reaction is the vaccine causing molecular mimicry. This molecular mimicry is then followed by an abnormal immune response culminating in uveitis and ocular damage. Although the literature suggests this patient to be at higher risk of developing rheumatoid arthritis and type 1 diabetes because of the presence of the HLA-DR4 gene, it was the vaccine-induced uveitis that elicited the closer scrutiny resulting in the discovery of her HLA type [35]. In this case, an important consequence of this finding would be the heightened care that can be provided by the physicians responsible for her care, thereby preventing unnecessary morbidity or mortality.

Another study involving a 27-year-old female with systemic lupus erythematosus after administration of the hepatitis B vaccine echoes the above sentiment [28]. In this study, the woman developed lupus nephritis after receiving the hepatitis B vaccine and this prompted researchers to study her HLA haplotype. What they found was the presence of alleles typical of both systemic lupus erythematosus and Sjogren's syndrome, leading them to conclude that the hepatitis B vaccine exacerbated an existing autoimmune disorder or triggered its emergence [28]. Their findings strengthen the fundamental argument of this paper and open the door to further research linking adverse vaccination reactions to underlying autoimmune diseases. Ultimately, the hope of this review is to help establish this link so that more patients can receive a timely diagnosis and treatment for their autoimmune diseases.

Another vaccine triggered condition is called the autoimmune/inflammatory syndrome induced by adjuvants (ASIA) [36]. The syndrome can be triggered by a multitude of factors, including exposure to chemicals (as in the Gulf war), in addition to vaccine adjuvants. The multitude of symptoms associated with this syndrome, the varied and possibly long time frame post-vaccination, and the low volume of reported cases make it challenging to make an ASIA diagnosis. Further research needs to be conducted to facilitate an easier and quicker diagnosis [36].

One case describes a patient who presented with unilateral uveitis, and shortly after, uveitis in the contralateral eye. After an extensive physical examination, the past medical history revealed the administration of the Shingrix vaccine within the week of eye pain presentation. Ophthalmic examination revealed granulomatous uveitis leading to the patient’s diagnosis of uveitis sarcoidosis presumably initiated after the administration of the Shingrix vaccination [22]. Sarcoidosis may present with uveitis as a systemic manifestation. The new Shingrix vaccination contains a new adjuvant, AS01B, which might be contraindicated in autoimmune patients due to the combination of adjuvants saponin-derived QS21 molecule and 3-O-desacyl-4'-monophosphoryl lipid A that heavily stimulate antigen presentation to T cells in the body [37]. Further studies can look into uveitis becoming a marker or indicator for patients to be given a more exhaustive workup for an autoimmune condition when presenting with uveitis as an initial symptom post-vaccination. 

## Conclusions

While no direct correlations can be made yet, the evidence in this paper warrants closer scrutiny of the field of autoimmune disorders and immunizations. The systematic overlap of uveitis in autoimmune diseases could mean a possible link between vaccine-induced uveitis and undiagnosed autoimmune disease. Researchers can, therefore, perform retrospective studies on vaccinated patients and investigate their HLA types, the occurrence of uveitis with a timeframe of the resolution, and associated autoimmune conditions. Additionally, retrospective studies could determine the patient’s immunization record, as well as presenting symptoms at the time of diagnosis of autoimmune disease.
